# Data for the Oxford Abdominal Aortic Aneurysm Study international survey of vascular surgery professionals

**DOI:** 10.1016/j.dib.2017.07.058

**Published:** 2017-07-27

**Authors:** Regent Lee, Amy Jones, Felicity Woodgate, Nicholas Killough, Kirthi Bellamkonda, Matthew Williams, Katherine Hurst, Lucy Fulford-Smith, Ismail Cassimjee, Ashok Handa

**Affiliations:** Nuffield Department of Surgical Sciences, University of Oxford, United Kingdom

## Abstract

As part of the Oxford Abdominal Aortic Aneurysm (OxAAA) Study, we conducted an international survey of vascular surgery professionals. One aspect of the survey is as published in the International Journal of Cardiology: “*International Opinion on Priorities in Research for Small Abdominal Aortic Aneurysms and the Potential Path for Research to Impact Clinical Management*”.

This Data-in-Brief article contains a detailed method for the conduct of this survey and additional original data. In this survey, we also provided vascular surgery colleagues with contemporary epidemiologic and surgical outcome data. This was followed by a *hypothetical* scenario whereby a patient had just been diagnosed with a small (40 mm) AAA and a novel biomarker predicted it to be fast growing in the coming years. We assessed the vascular professionals' perception of the patient's preference for management in this scenario, and their willingness to refer patients for a surgical trial that investigates the outcome of early versus late surgery in this setting. The survey then asked the vascular professionals to assume the role of the patient, and provided their own preferences in such a scenario.

**Specifications Table**

**Table:** 

Subject area	Cardiovascular Surgery
More specific subject area	Vascular Surgery
Type of data	Summary statistics
How data was acquired	Through an online survey, constructed using Google Form.
	The active link to the survey can be found at: http://tinyurl.com/OxAAASurveyInternational
Data format	Analysed
Experimental factors	N/A
Experimental features	Summary statistics of each category was performed
Data source location	Nuffield Department of Surgical Sciences, University of Oxford
Data accessibility	The raw data (in.xls format) can be downloaded from the Oxford University Website for the Oxford Abdominal Aortic Aneurysm Study.
	https://www.nds.ox.ac.uk/research/oxaaa
Related research article	International Opinion on Priorities in Research for Small Abdominal Aortic Aneurysms and the Potential Path for Research to Impact Clinical management – Iint J Cardiol 2017-1270 (in press) [1]

**Value of the data**

• In view of the lack of empirical evidence of the benefit of early surgery for patients with abdominal aortic aneurysms (AAA) that *will* be fast growing (as predicted by a novel biomarker), there is no basis upon which vascular professionals can make an evidence based recommendation. To reach a clinical decision in such a scenario, emphasis will be placed on the patient's values and preferences. Doctors sometimes make clinical decisions based on what they think patients prefer. This survey explored the vascular professionals’ perception of patients’ preferences.
• Patients often rely on surgeon's recommendations for their management of a AAA. In this hypothetical scenario, the absence of empirical evidence will result in clinicians’ equipoise on the best recommendation, which should be resolved by a clinical trial. This survey provides data on the vascular colleagues’ willingness to refer patients to a clinical trial.
• When designing a clinical trial, it is important to address the outcome measures that are important to the patients. Our data allows comparison between the vascular professionals’ perception of patient preferences against the actual preferences stated by the patients.

## 1 Data

**Table:** 

**Survey question**	**Responses (of 277)**
**Proportion of vascular professionals who thought that patients view AAA as a “disease”**	84%
**Proportion of vascular professionals who thought that patients get anxious around the time of AAA surveillance**	72%
**Proportion of vascular professionals who thought patients are:**	
“Very preoccupied” with the size of their AAA	35%
“Somewhat preoccupied” by the size of their AAA	35%
“Slightly preoccupied” by the size of their AAA	27%
“Not at all preoccupied” by the size of their AAA	3%
**Proportion of vascular professionals who thought the patient feels “cured” by AAA surgery**	84%
**In the*****hypothetical*****scenario where a biomarker predicts the small AAA to be fastgrowing** :	
Proportion who thought patients would prefer to have surgery early	58%
Proportion who thought patients would prefer to delay surgery	26%
Proportion who were unsure what patients would prefer	17%
**Amongst those vascular professionals who thoughts patients would prefer to have surgery early in the above hypothetical scenario:**	
Proportion who thought patients would prefer to have endovascular repair (EVAR)	55%
Proportion who thought patients would prefer to have open surgical repair (OSR)	14%
Proportion who thought patients would follow the surgeon's advice regarding the type of surgical repair	33%
**In the above hypothetical scenario, would the vascular professional consider referring the patient to a clinical trial to test the benefit of early surgery:**	
“Yes, definitely”	48%
“Maybe”	42%
“No, definitely not”	10%
**What vascular professionals thought was the most important outcome for the patient in such as clinical trial:**	
“To make the patients live longer”	40%
“To reduce the patients’ anxiety about their AAA”	42%
“To improve the patients’ quality of life“	18%

## 2 Experimental design, materials and methods

This online survey was constructed using Google Forms, and was delivered in two phases. The first phase was during the European Society of Vascular and Endovascular Surgery Annual Conference 2016 (Copenhagen), where we distributed 1500 copies of invitation flyers at the conference. The second phase was conducted during November 2016 and April 2017 as an online campaign.

In order to reach out to Vascular Surgeons internationally, the lead author (R Lee) searched the LinkedIn director for “vascular surgeon, vascular surgery” in different continents using the advanced search parameters in LinkedIn. This was the second phase of the survey. LinkedIn policies allow users to search and connect with others within 2nd or 3rd degree connections. (i.e. the user can only connect with someone who is connected to an existing connection of the user 2nd degree connection), or another user connected to the 2nd degree connection (3rd degree connection).

Using this approach, R Lee sent connection requests to more than ~ 7000 vascular surgeons worldwide. (The total number of connection requests sent by R Lee was likely in excess of this, but LinkedIn caps the total sent connection displayed at “5000”. We estimate that over 7000 connection requests were sent during the whole survey period). Out of these connections requests, about 2000 vascular surgeons connected with R Lee.

R Lee sent individual emails to each connected vascular surgery professional to invite them to take part in the survey, and received 254 responses as of the 24th of April, 2017. Including the 23 responses that were received during the first phase, the totally number of responses is 277.
