# Supplementary material for: Data for the Oxford Abdominal Aortic Aneurysm Study international survey of vascular surgery professionals
Source: Data Brief. 2017 Jul 27;14:298–301. doi: 10.1016/j.dib.2017.07.058 (PMC5540697; doi:10.1016/j.dib.2017.07.058)
Supplement: Supplementary file 1 — Supplementary material [file mmc1.docx]

16^th^ June 2017

Dear Editor,

**RE: DIB-D-17-00539R1**

**Vascular Professionals’ Perception of Patient Preferences in the Management of Small Abdominal Aortic Aneurysms Guided by Novel Biomarkers**

I confirm that the authors whose names are listed in this manuscript have NO affiliations with or involvement in any organization or entity with any financial interest (such as honoraria; educational grants; participation in speakers’ bureaus; membership, employment, consultancies, stock ownership, or other equity interest; and expert testimony or patent-licensing arrangements), or non-financial interest (such as personal or professional relationships, affi liations, knowledge or beliefs) in the subject matter or materials discussed in this manuscript.

Sincere yours

Regent Lee

Corresponding Author
